# Patchy Striatonigral Neurons Modulate Locomotor Vigor in Response to Environmental Valence

**DOI:** 10.1101/2025.02.28.640838

**Published:** 2025-05-12

**Authors:** Sarah Hawes, Bo Liang, Braden Oldham, Breanna T. Sullivan, Lupeng Wang, Bin Song, Lisa Chang, Da-Ting Lin, Huaibin Cai

## Abstract

Spiny projection neurons (SPNs) in the dorsal striatum play crucial roles in locomotion control and value-based decision-making. SPNs, which include both direct-pathway striatonigral and indirect-pathway striatopallidal neurons, can be further classified into subtypes based on distinct transcriptomic profiles and cell body distribution patterns. However, how these SPN subtypes regulate spontaneous locomotion in the context of environmental valence remains unclear. Using *Sepw1* - *Cre* transgenic mice, which label a specific SPN subtype characterized by a patchy distribution of cell bodies in the dorsal striatum, we found that these patchy striatonigral neurons constrain motor vigor in response to valence differentials. In a modified light/dark box test, mice exhibited differential walking speeds between the light and dark zones. Genetic ablation of these patchy SPNs disrupted restful slowing in the dark zone and increased zone discrimination by speed. *In vivo* recordings linked the activity of these neurons to zone occupancy, speed, and deceleration, with a specific role in mediating deceleration. Furthermore, chemogenetic activation of patchy SPNs—and optical activation of striatonigral neurons in particular—reduced locomotion and attenuated speed-based zone discrimination. These findings reveal that a subtype of patchy striatonigral neurons regulates implicit walking speed selection based on innate valence differentials.

## Full Text Availability

The license terms selected by the author(s) for this preprint version do not permit archiving in PMC. The full text is available from the preprint server.

