# Supplementary material for: Patchy Striatonigral Neurons Modulate Locomotor Vigor in Response to Environmental Valence
Source: bioRxiv. 2025 Mar 6:2025.02.28.640838. Preprint. [Version 1] doi: 10.1101/2025.02.28.640838 (PMC11908127; doi:10.1101/2025.02.28.640838)
Supplement: Supplementary file 1 [file media-1.pdf]

Patchy Striatonigral Neurons Modulate Locomotor Vigor in Response to Environmental Valence

Sarah Hawes[1], Bo Liang[2, 3], Braden Oldham[1], Breanna T. Sullivan[1], Lupeng Wang[1], Bin Song[1], Lisa Chang[1], Da-Ting Lin[2, 4, *], and Huaibin Cai[1,5 *]

Supplementary Figures and Figure legends

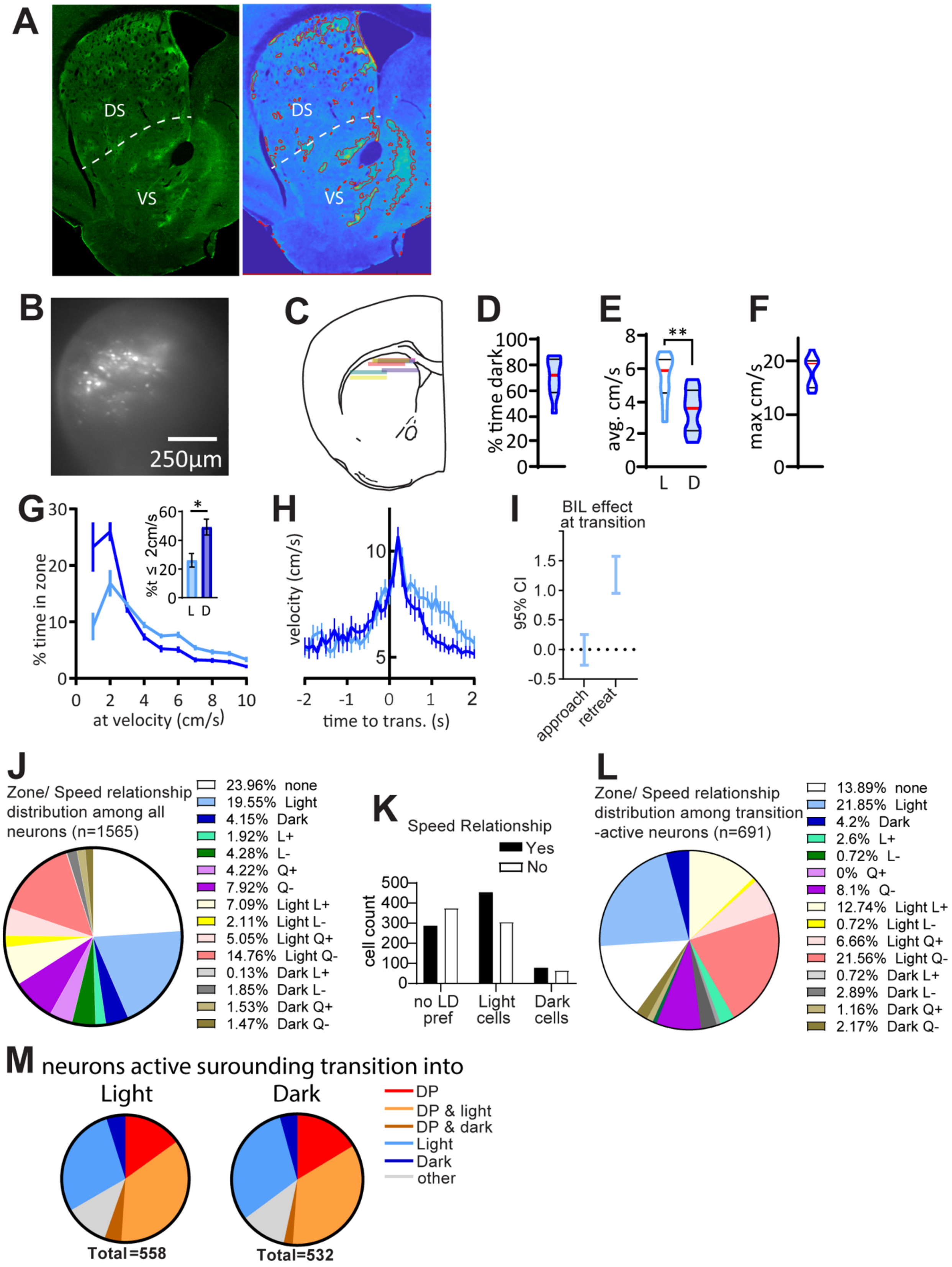
A
DS
VS
B
250µm
C
D
% time dark
E
**
avg. cm/s
L D
F
max cm/s
G
% time in zone
at velocity (cm/s)
%t ≤ 2cm/s
*
L D
H
velocity (cm/s)
time to trans. (s)
I
BIL effect at transition
95% CI
approach
retreat
J
Zone/ Speed relationship distribution among all neurons (n=1565)
23.96% none
19.55% Light
4.15% Dark
1.92% L+
4.28% L-
4.22% Q+
7.92% Q-
7.09% Light L+
2.11% Light L-
5.05% Light Q+
14.76% Light Q-
0.13% Dark L+
1.85% Dark L-
1.53% Dark Q+
1.47% Dark Q-
K
Speed Relationship
Yes
No
cell count
no LD pref
Light cells
Dark cells
L
Zone/ Speed relationship distribution among transition -active neurons (n=691)
13.89% none
21.85% Light
4.2% Dark
2.6% L+
0.72% L-
0% Q+
8.1% Q-
12.74% Light L+
0.72% Light L-
6.66% Light Q+
21.56% Light Q-
0.72% Dark L+
2.89% Dark L-
1.16% Dark Q+
2.17% Dark Q-
M neurons active surounding transition into
Light
Dark
DP
DP & light
DP & dark
Light
Dark
other
Total=558
Total=532

Fig. S1. MOR1 thresholding, Scope-mounted Light/Dark box behavior and Ca^{2+} imaging supplemental. (A) Patch ablation was assessed by MOR1-positive area measurements. Example dorsal striatal (DS) and ventral striatal (VS) hemisection stained for MOR1 (left) alongside thresholded image generated in MATLAB (right) used for quantifying MOR1-positive territories. **(B)** example field of view through GRIN lens. **(C)** schematic of lens positions in imaged mice. **(D-L)** n=10 scope-mounted mice. **(D)** % time in dark. **(E)** average speed. Wilcoxon L vs D **p=0.0098. **(F)** maximum speed. **(G)** speed distribution normalized to zone. %time ≤2cm/sec (bar graph) Wilcoxon L vs D *p=0.0137. **(H)** transition speed. **(I)** GLME fixed effects 95% CI (cm/s) in brackets. Approach speed is unchanged by BIL [0.2506, -0.26603]. Retreat speed is increased by BIL [1.576, 0.9505]. **(J)** distribution of zone and/or speed relationships among all recorded neurons. **(K)** relationship between zone preference and speed encoding χ^2 (3, n=532) =78.63, p<0.0001 **(L)** distribution of zone and/or speed relationships among all transition-active neurons. **(M)** distribution of zone and/or deceleration relationships among all transition-active neurons.

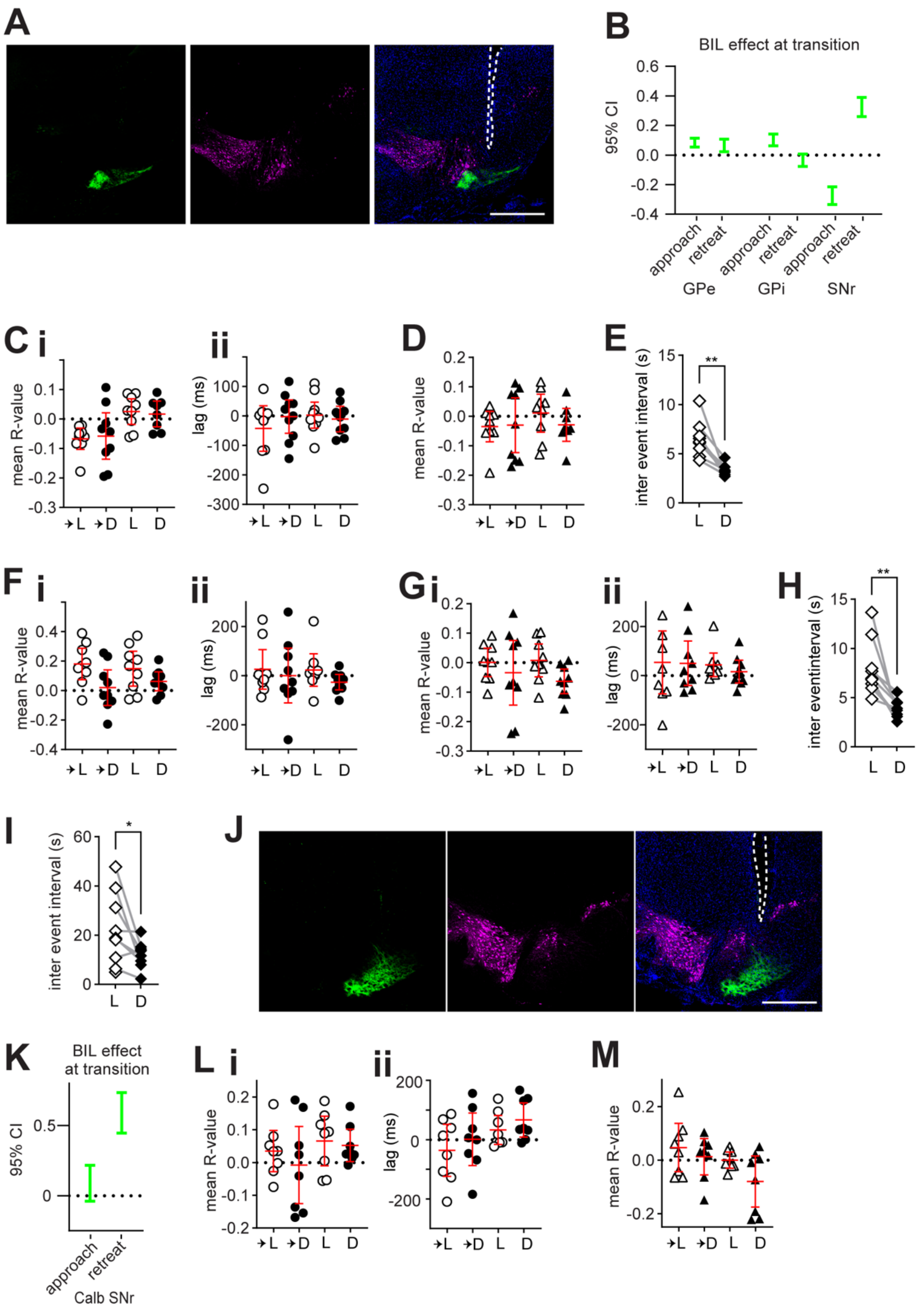

Fig. S2. Fiber photometry supplemental. (A-G) n=8 GPe-implanted, 9 GPi-implanted *Sepw1*-Cre mice. **(A)** transition BIL effect on patchy SPN efferents. GLME fixed effects 95% CI

(%ΔF/F) in brackets. **(B-D)** Analysis of patchy SPN efferents in GPe. 95% CI in red. **(Bi)** for all acceleration events with significant ($p<0.05$) ΔF/F cross-correlation to speed, mean R value per mouse, **(ii)** mean Xcorr lag per mouse. **(C)** for all acceleration events with significant ($p<0.05$) ΔF/F cross-correlation to acceleration, mean R value per mouse. **(D)** event frequent in either zone, paired t-test **p=0.0018 **(E-G)** Analysis of patchy SPN efferents in GPi. 95% CI in red. **(Ei)** for all acceleration events with significant ($p<0.05$) ΔF/F cross-correlation to speed, mean R value per mouse, **(ii)** mean Xcorr lag per mouse. **(Fi)** for all acceleration events with significant ($p<0.05$) ΔF/F cross-correlation to acceleration, mean R value per mouse. **(ii)** mean Xcorr lag per mouse. **(G)** event frequent in either zone, paired t-test **p=0.0035 **(H)** n=9 GRIN-implanted *Sepw1*-Cre mice. From net fluorescence collected in dorsal striatum, event frequent in either zone, paired t-test *p=0.0373 **(I-K)** n= 8 SNr-implanted Calb1 Cre mice. **(I)** transition BIL effect on matrix efferents. GLME fixed effects 95% CI (%ΔF/F) in brackets. **(J-K)** Analysis of matrix efferents in SNr. 95% CI in red. **(Ji)** for all acceleration events with significant ($p<0.05$) ΔF/F cross-correlation to speed, mean R value per mouse, **(ii)** mean Xcorr lag per mouse. **(K)** for all acceleration events with significant ($p<0.05$) ΔF/F cross-correlation to acceleration, mean R value per mouse.

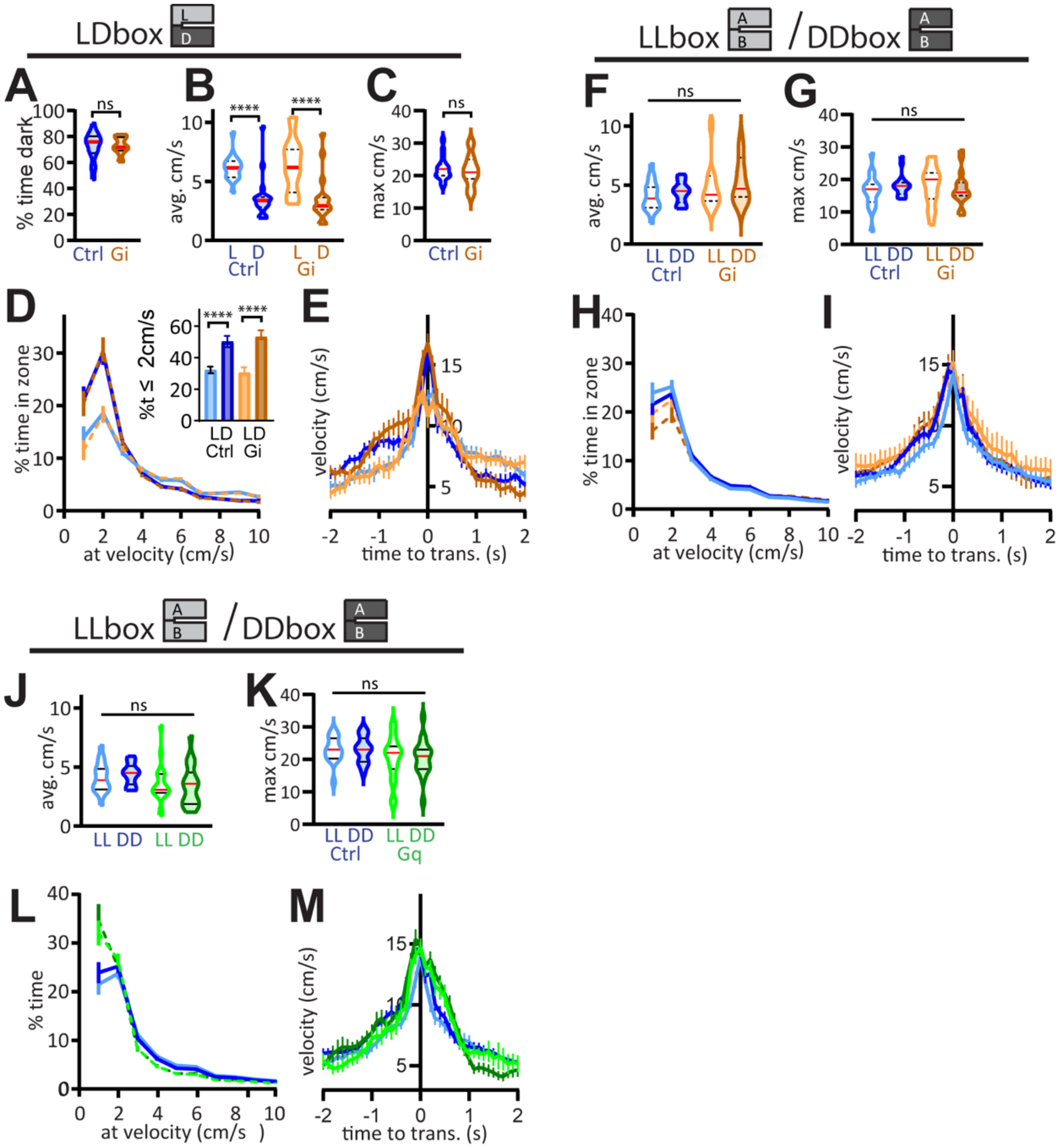

Fig. S3. Chemogenetics supplemental. (A-E) Gi-mice L/D box, n=17 Ctrl and 15 Gi mice. **(A)** % time in the dark. Mann-Whitney Ctrl vs Gi p=0.8232. **(B)** Average speed. 2way RM ANOVA: group F (1, 30) = 0.03006, p>0.05; light F (1, 30) = 134.3, p<0.0001. **(C)** Maximum speed. 2-tailed Mann Whitney, p=0.715. **(D)** Speed distribution normalized to zone. For %time ≤2cm/sec (bar graph) paired t-test L vs D Ctrl p<0.0001, Gi p<0.0001, unpaired t-test Ctrl vs Gi in Light p>0.05, in Dark p>0.05. **(E)** Transition speed. GLME fixed effects 95% CI (cm/s) in brackets.

Approach speed is increased by BIL [1.0773, 0.59214] and unchanged by Gi [-1.0473, 1.0196]; Retreat speed is increased by BIL [1.0562, 1.486] and unchanged by Gi [-1.3062, 0.92042]. GLME by group shows approach speed is increased by BIL for control [1.9076, 1.3185] and Gi [2.2463, 1.5553]. **(F-I) Gi-mice LL/DD box**. n= mouse (16 Ctrl, 13 Gi). **(F)** Average speed. 2way RM ANOVA: lighting $F(1, 27) = 3651$ $p=0.0667$, group $F(1, 27) = 2.994$ $p>0.05$. **(G)** Maximum speed. 2way RM ANOVA: lighting $F(1, 27) = 0.05506$ $p>0.05$, group $F(1, 27) = 1.402$ $p>0.05$. **(H)** Speed distribution normalized to zone. For %time ≤2cm/sec (bar graph) Wilcoxon LL vs DD Ctrl $p>0.05$, Gi $p=0.0105$, Mann-Whitney Ctrl vs Gi in Light $p>0.05$, in Dark $p>0.05$. **(I)** Transition speed. GLME fixed effects 95% CI (cm/s) in brackets. Approach speed is slightly increased by BIA [0.85304, 0.42385], unchanged by Gi [-0.25536, 1.985]; Retreat speed is slightly increased by BIA [0.31052, 0.76904], unchanged by Gi [-0.73445, 1.7233]. **(J-M) Gq-mice LL/DD box**. n= mouse (16 Ctrl, 19 Gq). **(J)** Average speed. 2way RM ANOVA: lighting $F(1, 33) = 2.676$ $p>0.05$, group $F(1, 33) = 1.343$ $p>0.05$. **(K)** Maximum speed. 2way RM ANOVA: lighting $F(1, 33) = 2.683$ $p>0.05$, group $F(1, 33) = 0.0008$ $p>0.05$. **(L)** Speed distribution normalized to zone. For %time ≤2cm/sec Wilcoxon LL vs DD Ctrl $p>0.05$, Gq $p>0.05$, Mann-Whitney Ctrl vs Gq in LL $p=0.0441$, in DD $p=0.0288$. **(M)** Transition speed. GLME fixed effects 95% CI (cm/s) in brackets. Approach speed is increased by BIA [0.85304, 0.42385], unchanged by Gq [-1.1387, 0.89713]; Retreat speed is increased by BIA [0.31052, 0.76904], unchanged by Gq [-1.5338, 0.69961].
